# Egocentric network characteristics of people who inject drugs in the Chicago metro area and associations with hepatitis C virus and injection risk behavior

**DOI:** 10.1186/s12954-022-00642-4

**Published:** 2022-06-02

**Authors:** Mary Ellen Mackesy-Amiti, Joshua Falk, Carl Latkin, Maggie Kaufmann, Leslie Williams, Basmattee Boodram

**Affiliations:** 1 Division of Community Health Sciences, School of Public Health, University of Illinois at Chicago, 1603 W. Taylor St, Chicago, IL 60612 USA; 2 Advanced Cyberinfrastructure for Education and Research (ACER), Office of the Vice Chancellor of Innovation, University of Illinois at Chicago, 728 W. Roosevelt Rd., 215A RRB, Chicago, IL 60607 USA; 3 Department of Health, Behavior, and Society, Bloomberg School of Public Health, Johns Hopkins University, 615 N. Wolfe St, Baltimore, MD 21205 USA; 4 Community Outreach Intervention Projects, School of Public Health, University of Illinois at Chicago, 1603 W. Taylor St, Chicago, IL 60612 USA

**Keywords:** Hepatitis C, Injection drug use, Risk behavior, Egocentric networks, People who inject drugs, Injection network, Support network, Network structure, Geography

## Abstract

**Background:**

Hepatitis C (HCV) infection has been rising in the suburban and rural USA, mainly via injection-based transmission. Injection and sexual networks are recognized as an important element in fostering and preventing risky behavior; however, the role of social support networks has received somewhat less attention.

**Methods:**

Using baseline data from an ongoing longitudinal study, we examined the composition and structure of injection drug use (IDU), sex, and social support networks of young people who inject drugs (aged 18–30) and their injection network members. Lasso logistic regression was used to select a subset of network characteristics that were potentially important predictors of injection risk behaviors and HCV exposure.

**Results:**

Several measures of IDU, sexual, and support network structure and composition were found to be associated with HCV exposure, receptive syringe sharing (RSS), and ancillary equipment sharing. Gender and sexual relationships were important factors for all risk behaviors. Support network characteristics were also important, notably including a protective effect of majority Hispanic support networks for RSS and HCV exposure. Both IDU network residence heterogeneity and support network geography were associated with injection equipment sharing.

**Conclusions:**

The associations of IDU and support network geography with equipment sharing highlight the need to extend harm reduction efforts beyond urban areas. Greater understanding of support network influences on risk behavior may provide important insights to strengthen the benefits of harm reduction. In considering the probability of HCV transmission, it is important to consider setting and network structures that promote propagation of risk.

**Supplementary Information:**

The online version contains supplementary material available at 10.1186/s12954-022-00642-4.

## Introduction

Injection drug use (IDU) is the primary mode of hepatitis C (HCV) transmission in developed countries such as the USA [1], with an estimated 67% of all HCV infections attributable to IDU in 2019 [2]. Fueled by the opioid epidemic, HCV incidence has increased fourfold between 2008 and 2019 [3], primarily driven by young people who inject drugs (PWID) from non-urban areas [4–9], and HCV-related deaths have now surpassed the total combined deaths from 60 other infectious diseases in the USA, including HIV and late-stage HIV-related illness [10].

Social network factors have been studied for their role in drug equipment sharing behavior (reviewed in De et al. [11]) and HIV risk [12–14], with fewer studies focused on HCV among US PWID [15, 16]. To achieve the World Health Organization’s (WHO) goal of reducing new chronic infections by 90% and mortality by 65% by 2030 [17] in the USA, innovative strategies are needed, as the USA continues to lag behind most high-income countries in achieving this goal [18]. HCV transmission through IDU necessarily involves close personal interactions among PWID. In addition to an injection risk network that is linked by syringe-sharing behaviors, most PWID are embedded in other (e.g., sexual and support) networks that may overlap with their IDU networks to varying degrees. Characteristics and structure of these networks can affect the likelihood of engaging in risky behaviors that are associated with HCV risk [11, 19–22] and provide insight into network-based mitigation interventions.

Using baseline data from an ongoing longitudinal egocentric network study of young PWID and their injection, sexual, and support networks from a large metro area, we report on two gaps in the current body of research. First, building on previous work by our team [15, 23], to our knowledge this is the first study to report on the injection, support, and sexual network characteristics and structure of the emerging population of young, predominantly non-Hispanic white suburban PWID from a large metropolitan area. Second, while network factors have been extensively examined in relation to HIV risk and interventions, there is a dearth of studies that have focused on hepatitis C; no prior study to our knowledge has simultaneously examined the role of injection, sexual, and support network size, attributes, and structure in association with injection risk behaviors and HCV infection in this population. Specifically, we examine associations between risky injection behavior (e.g., receptive syringe sharing) and aggregate measures of composition and structure of egocentric injection, support, and sexual activity networks among young urban and suburban PWID. Our study will provide a better understanding of the network factors among young PWID, the population primarily driving HCV incidence in the USA, toward achieving the World Health Organizations goal of HCV elimination [17].

## Methods

The alter–ego study is an ongoing (2017-) longitudinal network-based study of young (aged 18–30) PWID (egos) and their injection network members (alters). Baseline interview data were used for the analysis.

### Eligibility

**Fig. 1:**
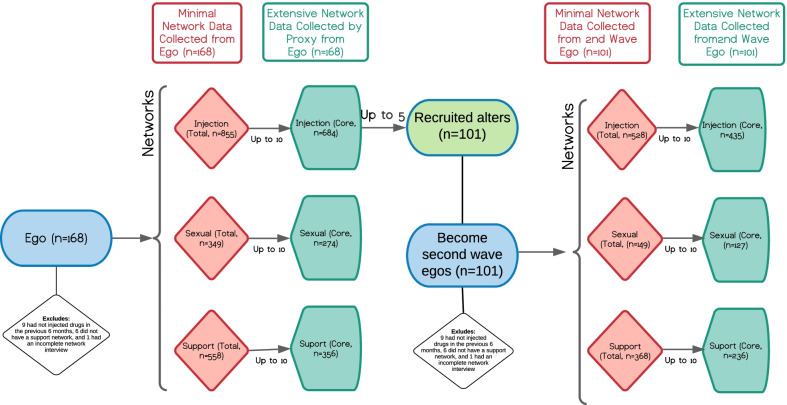
Ego and alter sample generation schema

To be eligible, ego participants (i.e., initial participants who were asked to recruit their network members) had to be (1) 18–30 years old, (2) injected drugs at least once in past 30 days, (3) willing to recruit PWID ≥ 18 years old at baseline with whom they injected drugs in the past 6 months (i.e. , injection network members), (4) willing to be tested for HIV and HCV, and (5) residing in the Chicago Metropolitan Statistical Area in the past 12 months. The injection alters were eligible if they were (1) ≥ 18 years old and (2) had injected drugs with the ego in the past 6 months. Current IDU was verified by experienced study staff checking for injection stigmata and, if questionable, using a standardized procedure to evaluate participant knowledge of the injection process. Age was verified with a driver’s license or a state ID card. Project staff offered to assist those without identification in obtaining it. Figure 1 shows the sample generation process.

### Ego recruitment

The study was conducted at two field sites of a community outreach center located in Chicago, Illinois, USA, that has been providing services (e.g., syringe service programs; and HIV and HCV counseling, testing, and case management) and conducting research on people who use illicit substances for over 30 years. The field sites are located in areas that have rates above the city’s average for HIV/AIDS, sexually transmitted infections, viral hepatitis, and arrests for drug-related offenses and attract both urban and suburban PWID. We recruited most egos from the syringe services program (SSP) at these field sites. In addition, SSP-recruited participants were screened to ascertain if they obtained syringes at the SSP for other people who reside in the suburbs. Those who did were offered a coupon to refer to the study an age-eligible peer who did not use the SSP or purchase/use drugs in Chicago. To encourage peer-recruited PWID to participate, we used a mobile outreach van staffed with an interviewer/phlebotomist, to conduct data collection off-site near the recruit’s residence or other mutually agreed upon locations. Alternative outreach methods targeting non-SSP suburban PWID included direct recruitment in drug market areas and at community health events using an outreach van, flyers posted at community-based organizations serving PWID, and social media and other online ads. Screening and enrollment of non-SSP PWID from drug market areas were done by indigenous field staff with extensive experience working in these areas and recruiting for similar studies.

### Alter recruitment

At their baseline visit, we asked each ego participant to recruit up to five alters in their core injection network, defined as people with whom they injected drugs at least once in the past six months, using recruitment coupons that provided information about the study and were linked to the recruiting ego via alphanumeric code. Coupons could only be redeemed by alters named by an ego participant during their survey. Data collection from alters was required to occur within 6 months of the ego’s baseline visit.

### Procedures

All participants (egos and alters) completed a process of informed consent. All study procedures were approved by an Institutional Review Board at the University of Illinois at Chicago. Participants received compensation of $20/hour for the interview. Most participants completed the survey within a 2.5 h session that included a break (average $50). In addition to hourly compensation for interviews, egos were reimbursed $10 for each alter enrolled.

All participants received HIV and HCV testing and counseling. All services (e.g., SSP; HCV and HIV testing, counseling, and case management; and linkage to medical care) were made freely available to all PWID screened, regardless of study enrollment.

Participants completed a baseline computer-assisted interviewer-administered questionnaire, including background demographics, substance use, HCV testing, injection-related behaviors, and other measures. Egonet data were then collected from participants using GENSI software [2016] [24]. The survey is touch screen enabled, and the participant can tactilely participate in the collection of network data via the “binning” of software-generated nodes which represent the members of their IDU, sex, and support networks, with the interviewer. The interview begins with the participant being asked to identify and generate their injection, sexual, and social support networks. They are first asked to generate their total networks (anyone they have injected with, had sex with, or received support from at least once in the past six months). They are then asked to report the gender, age, primary method of drug use and substance, race/ethnicity, and county of residence for each. For social support network members, participants are asked what their relationship is to the network member (friend, relative, etc.). Once they have generated the total network, they are asked to identify which of the members are in their core injection networks, core social support networks, and core sex networks (see definitions below). The remainder of the survey focuses on these core networks. Starting with the participants’ IDU core, they are asked questions related to each member and are asked to “bin” the bubbles that represent their network members in the appropriate answer bin. They are also asked how well the members of their 3 networks know each other to collect the strength of tie for each relationship.

### Network survey

#### Injection network

Participants were asked to identify people who they used drugs with in the past six months (total injection network). Among those, they were then asked to identify a core injection network of up to ten people who they used drugs with more than once in the past six months. Data on the injection network were collected by proxy for the total and core injection network, and directly from up to five alters identified and recruited by egos.

#### Support network

Participants were asked to identify people who provided support to them in the past six months (total support network). This included “anyone you could talk to about things that are personal and private or get advice from if a situation came up” (personal support), and “anyone that would let you stay at their place if needed” (shelter support). Among their support network, they were then asked to identify a core support network of up to ten people who provided the most support in the past six months.

#### Sex network

Participants were asked to identify people they had sex with in the past six months, including vaginal, anal, or oral sex. Among these, they were then asked to identify a core sex network of up to ten people who the participant had sex with more than once in the past six months.

### Individual measures (ego and alters)

#### Demographics

Self-reported gender, age, race, and Hispanic/Latinx ethnicity were collected. Gender was reported as male, female, transgender, or other. Multiple categories could be selected for race; ethnicity was indicated separately as Hispanic/Latinx or not Hispanic/Latinx. Race and ethnicity were also combined to create an indicator variable with mutually exclusive categories: non-Hispanic white (white only), non-Hispanic Black (including mixed), Hispanic (any race), and non-Hispanic other race. Employment was assessed based on responses to the question “During the last 6 months, did you receive any money from any of these sources?” Participants who indicated that they received money from a regular job (full or part-time) or self-employment were classified as employed. Egos were similarly asked to provide sociodemographic information on their alters as part of the network interview.

#### HIV and HCV status

Rapid HIV and HCV antibody (Orasure Technologies, 2004, 2011) testing was used to determine prevalence of exposure (past or current infection) for egos and recruited alters.

#### Syringe and equipment sharing

Injection behavior was assessed on a 5-point Likert-type scale (never, less than half the time, about half the time, more than half the time, or always). Questions assessed receptive syringe sharing (RSS) (“When you shot-up in the last 6 months, how often did you use a syringe that you know for sure had been used before by someone else?”), equipment sharing (“When you injected drugs in the last six months, how often did you use any of the following items with other people? (a) drawn from the same cooker, (b) used the same cotton, (c) used the same rinse water”), and backloading (“In the last six months, how often did you inject with a syringe AFTER someone else has squirted drugs into it from their syringe?”). Equipment sharing was coded as the maximum of the three items (cookers, cottons, water). Each of these measures was dichotomized to indicate any behavior in the past six months.

### Network measures

Summary measures of ego network size, composition, and structure were computed for the IDU, sex, and support networks, which are defined and described in an additional table (see Additional file 1: Table S1). Ego–alter tie strength was based on frequency of contact, assessed by asking “How often do you talk to or see this person?” with options on a 6-point scale from every day to less than once a year. The last 3 categories were collapsed into once a month or less to create a 4-point scale. Alter–alter tie strength was based on the ego’s assessment of how well the two people knew each other, on a 4-point scale with anchors 1 = “casual acquaintance” and 4 = “very close relationship.”

All network measures were computed using NetworkX version 2.4 [25], except modularity, which was computed using the igraph R package version 1.2.5 [26]. Modularity was computed by finding the network partition that maximizes Newman’s Q. Hierarchy was implemented following Eq. 2.9 of Burt [27]. For measures that rely on distance rather than tie strength (for example, betweenness and closeness centrality), the scale was reversed, so that strongest ties were coded with a distance of 1 and weakest ties with a distance of 4. Average and local clustering, average distance, centrality, centralization, constraint, effective size, efficiency, and hierarchy were all computed both with real-valued edge weights based on tie strength and with binary edge weights based on presence/absence of a tie. Centralization measures and hierarchy are undefined when ego has degree one; these were recoded to zero for analysis.

#### Imputation of missing ties

Missing ties and tie strengths were imputed using sklearn version 0.22.1 [28]. For ego–alter tie strength, a random forest classifier was trained using all available ego–alter tie strengths, with ego sex, alter sex, and relationship types as features. Tie strength was randomly sampled for missing data according to the predicted probability distribution from the classifier. For alter–alter ties and tie strengths, a random forest classifier was trained using all alter–alter networks without missing data, with the sex of each alter and the relationship types between ego and each alter as features. Tie presence and strength were randomly sampled according to the predicted probability distribution from the classifier.

#### Covariates and outcomes

Covariates included 40 injection network variables, 40 support network variables, and 2 sex network variables, plus ego demographic variables: age, sex (female vs. male), and binary indicators of white, Black, and Hispanic. Risk behavior outcomes were binary indicators of RSS, equipment sharing, and backloading in the past six months.

### Sample

Figure 1 summarizes the unique participants (egos and alters). Of the 177 egos who completed the baseline study visit and provided proxy data on their total and core injection, sexual, and support networks, 9 were excluded: 6 did not have any support network, and 3 had missing data on key variables. Of the 117 alters who were recruited into the study and completed the baseline study visit in person, 16 were excluded: 9 had not injected drugs in the previous 6 months, 6 did not have a support network, and 1 had an incomplete network interview. In addition to providing data on relationship with egos, recruited alters also became second wave egos. The final analytic sample includes 168 ego and 101 alter (second wave ego) participants (total = 269).

### Analysis

Given the large number of network measures potentially associated with risk behavior, some of which are highly correlated with one another, we conducted adaptive lasso logistic regression (29, 30). The advantage of lasso regression is the k-fold cross-validation, which estimates prediction error by resampling to select potentially important predictors of HCV antibody positive status and injection risk behavior from a large set of network variables. Lasso regression is a supervised machine-learning method in which the goal is to obtain the subset of predictors that minimizes prediction error. The lasso procedure performs both variable selection and regularization, using shrinkage to select a subset of predictors, which prevents overfitting and encourages simple sparse models [31, 32]. Analyses were conducted using Stata (v. 16).

Adaptive selection in Stata uses cross-validation (CV) to select lambda (the shrinkage parameter), but multiple lassos are performed. In the first lasso, a lambda is selected, and penalty weights are constructed from the coefficient estimates. Then, these weights are used in a second lasso where another lambda is selected. Variables with zero coefficients are discarded after each successive lasso, and variables with nonzero coefficients are given penalty weights designed to drive small coefficient estimates to zero in the next step. CV is done by dividing the data randomly into folds. One fold is chosen and then a regression is fit on the other folds using the variables in the model for that lambda. With these new coefficient estimates, a prediction is computed for the data of the chosen fold. The mean squared error (MSE) of the prediction is computed. This process is repeated for the other folds. The MSEs from all folds are then averaged to give the value of the CV function. We repeated the analysis with 4 different random seeds and increased the number of folds until a result was replicated consistently.

HCV and risk behavior outcomes (RSS, equipment sharing, and backloading) were regressed on the 40 injection network variables, 40 support network variables, 2 sex network variables, ego age, sex (female vs. male or undetermined), and binary indicators of white, Black, and Hispanic.

#### Exploratory analysis

We conducted post hoc logistic regression analyses and computed predicted probabilities to explore the relationships between selected predictors and outcomes.

#### Sensitivity analysis

We conducted sensitivity analyses to compare the results using imputation with alternative strategies for missing data, including (1) listwise deletion and (2) imputation of ego–alter tie strengths only, treating missing alter–alter ties as nonexistent.

## Results

Table 1 shows the demographic characteristics of the 269 participants included in the analysis. One person identified as transgender; in calculating gender homophily of the network, they were treated as a third gender. HCV antibody positive prevalence was 18% among Hispanic participants, compared to 36% among non-Hispanic white, 39% among non-Hispanic Black, and 56% among participants of mixed or other race (*χ*^2^ = 11.30, *p* = 0.01). Thirty-four participants (12%) had incomplete network data due to a software malfunction or interviewer error early in the study period, resulting in a total of 45 ego–alter tie strengths (*n* = 16 participants) and 163 alter–alter ties (*n* = 27 participants) that were not recorded. Missing ties were imputed as described above.

**Table 1:** Characteristics of participants (*n* = 269)

	*n*	%
Gender		
Male	199	74%
Female	69	26%
Transgender	1	0.4%
Race/ethnicity		
NH white	162	60%
NH Black	18	7%
Hispanic (all races)	71	26%
NH other	18	7%
Age		
18–30	201	75%
31–40	47	17%
> 40	21	8%
Mean (SD)	30.1 (7.5)	
Range	[18–64]	
Residence		
Cook county	209	78%
Outer suburbs	60	22%
Employed^a^		
No	159	59%
Yes	110	41%
HCV antibody status^b^		
Positive	84	31%
Negative	173	64%
Receptive syringe sharing		
No	154	57%
Yes	115	43%
Shared cooker past 6 m		
No	73	27%
Yes	195	72%
Backload past 6 m		
No	169	63%
Yes	100	37%
Participant type		
Ego	171	64%
Alter	98	36%

^a^Received money from a regular job (full or part-time) or self-employment

^b^Baseline rapid antibody test; 12 missing due to COVID-19 interruption. Baseline HIV positive 1.2% (*n* = 3)

### Networks

Summary statistics for the IDU, support, and sexual network measures are presented in Table 2. Additional summary statistics for these three networks are reported in an additional table (see Additional file 2: Table S2). Mean IDU network degree was 3.9 (SD 2.4), while mean support network degree was 2.2 (SD 1.4). Among the 80% of participants with at least one sex partner (*n* = 216), degree of the sex network ranged from one to nine with a mean of 1.8 (SD 1.3), and mean multiplexity of sex partners was 1.9 (SD 0.7). Support networks had greater tie density, tie strength, and race/ethnicity homophily compared to injection networks.

**Table 2:** Summary statistics for injection, support, and sexual network measures (*n* = 269)

Variable	Injection				Support				Sexual^a^			
	Mean	SD	Min	Max	Mean	SD	Min	Max	Mean	SD	Min	Max
Size (degree)	3.94	2.4	1.0	10.0	2.17	1.4	1.0	10.0	1.80	1.3	1.0	9.0
Percent male	0.70	0.3	0.0	1.0	0.46	0.4	0.0	1.0	0.28	0.4	0.0	1.0
Percent white	0.62	0.4	0.0	1.0	0.66	0.4	0.0	1.0	0.60	0.4	0.0	1.0
Percent Black	0.13	0.3	0.0	1.0	0.10	0.3	0.0	1.0	0.10	0.3	0.0	1.0
Percent Hispanic	0.20	0.3	0.0	1.0	0.19	0.4	0.0	1.0	0.24	0.4	0.0	1.0
Percent employed	0.32	0.3	0.0	1.0	0.61	0.4	0.0	1.0	0.50	0.4	0.0	1.0
Percent reside in CC	0.73	0.4	0.0	1.0	0.61	0.4	0.0	1.0	0.61	0.4	0.0	1.0
Alter mean age	32.70	6.4	19.5	58.0	40.70	12.0	19.5	74.0	30.14	7.7	18.0	61.5
Gender homophily	0.58	0.3	0.0	1.0	0.41	0.4	0.0	1.0	0.04	0.2	0.0	1.0
Age homophily	0.54	0.4	0.0	1.0	0.50	0.4	0.0	1.0	0.60	0.5	0.0	1.0
Race/ethnicity Homophily	0.59	0.4	0.0	1.0	0.69	0.4	0.0	1.0	0.57	0.5	0.0	1.0
Residence homophily	0.69	0.4	0.0	1.0	0.60	0.4	0.0	1.0	0.61	0.4	0.0	1.0
Mean strength of ties	3.08	0.6	1.0	4.0	3.43	0.7	1.0	4.0	3.26	0.9	1.0	4.0
Tie density	0.85	0.2	0.3	1.0	1.59	0.6	1.0	3.0	0.89	0.2	0.2	1.0
Closeness centralization (bin.)	0.29	0.3	0.0	1.0	0.95	0.1	0.5	1.0	0.23	0.4	0.0	1.0
Closeness centralization (val.)	0.37	0.3	0.0	1.0	0.12	0.3	0.0	1.0	0.18	0.3	0.0	1.0
Closeness centrality (val.)	0.60	0.2	0.3	1.0	0.29	0.4	0.0	1.0	0.71	0.3	0.3	1.0
Constraint (bin.)	0.74	0.3	0.2	1.1	0.74	0.3	0.3	1.0	0.87	0.3	0.1	1.1
Constraint (val.)	0.72	0.3	0.3	1.6	0.93	0.2	0.3	1.1	0.86	0.3	0.1	1.5
Mean multiplexity	1.43	0.5	1.0	3.0	0.90	0.2	0.3	1.4	1.92	0.7	1.0	3.0
Efficiency (bin.)	0.57	0.3	0.1	1.0	0.70	0.3	0.1	1.0	0.90	0.2	0.2	1.0
Efficiency (val.)	0.69	0.2	0.2	1.0	0.78	0.2	0.2	1.0	0.93	0.2	0.2	1.0
Gender heterogeneity	0.27	0.2	0.0	0.5	0.21	0.2	0.0	0.5	0.02	0.1	0.0	0.5

Heterogeneity, Blau’s Index; bin, binary; val, valued; and CC, Cook County

^a^*N* = 216 (53 have no sex partners)

### Lasso regression

The results of the lasso regression analysis with 87 covariates are shown in Table 3. The out-of-sample deviance ratio is an estimate of the prediction performance of the model on a new sample relative to the null model. Consistent results were obtained with 50 folds for HCV status and 80 folds for risk behaviors. Being white was significant in the models for RSS and equipment sharing and was the only ego demographic variable selected.

**Table 3:** Penalized coefficients of variables selected in adaptive lasso regression

	Receptive syringe sharing	Equipment sharing	Backload	HCV positive
Demographics				
White	3.249	2.573	–	–
IDU network				
Mean age	–	0.977	–	1.099
Percent Hispanic	–	–	–	0.282
Gender homophily	–	0.560	–	–
Gender heterogeneity	1.539	–	6.102	–
Residence heterogeneity	–	1.829	–	–
Efficiency (binary)	–	0.613	–	–
Efficiency (valued)	0.093	–	–	–
Closeness centrality (valued)	0.403	–	–	–
Constraint (valued)	–	0.186	–	–
Support network				
Mean age	0.973	–	–	–
Percent Hispanic	0.444	–	–	0.476
Percent Cook County	–	0.575	–	–
Gender heterogeneity	–	–	–	0.266
Sex network				
Mean multiplexity	1.356	–	–	–
Lambda	0.003	0.021	0.002	0.004
Out-of-sample deviance ratio	0.086	0.064	0.016	0.084
CV mean deviance	0.086	1.004	1.299	1.158
*N*	269	268	269	257

RSS was negatively associated with valued measures of efficiency and closeness centrality in the injection network, and positively associated with injection network gender heterogeneity (mixed gender network) and sex partner multiplexity. Support network older age and greater proportion Hispanic were negatively associated with RSS. Equipment sharing was positively associated with injection network residence heterogeneity (mixed Cook and non-Cook county), and negatively associated with injection network age, gender homophily, efficiency (binary), and constraint (valued). Having a greater proportion of support network members who lived in Cook County was also negatively associated with equipment sharing. The only network variable predictor selected in the model for backloading was injection network gender heterogeneity which was positively associated with a sixfold greater likelihood of backloading. HCV antibody positive status was associated with older age of the IDU network, and negatively associated with percent Hispanic in both the IDU and support network. HCV positivity was also negatively associated with support network gender heterogeneity.

**Fig. 2:**
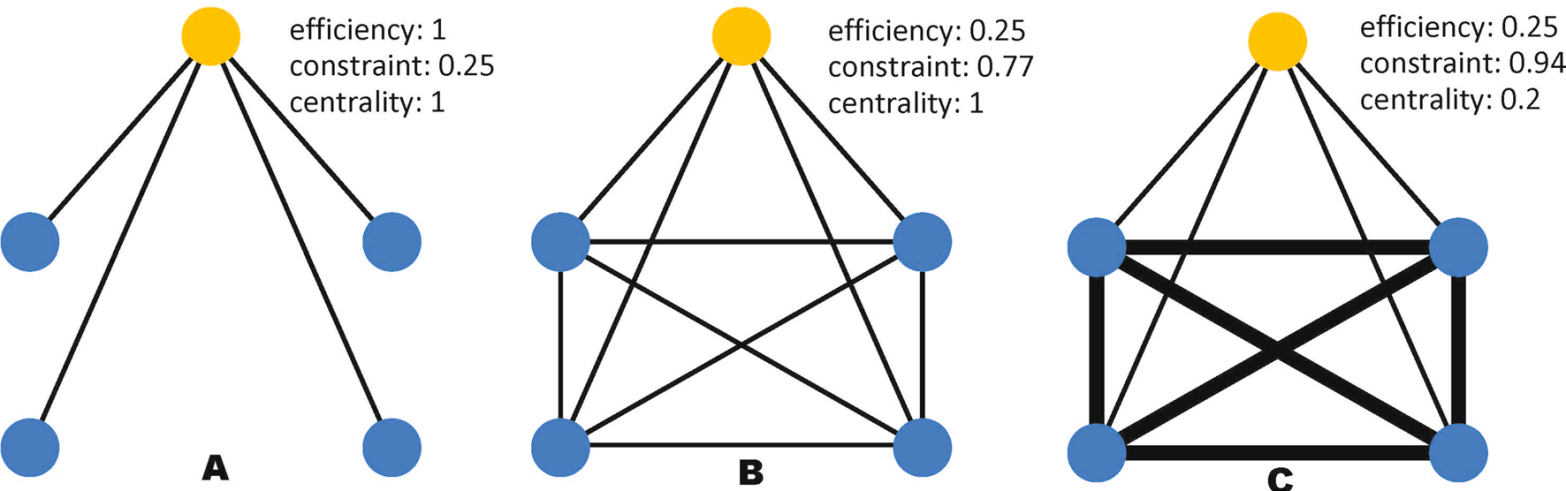
Egocentric network exemplars to illustrate measures of efficiency, constraint, and closeness centrality. In each graph, the top node in yellow represents ego, while the blue nodes below represent alters. In graphs **A** and **B** ties are unweighted. In graph **C**, strong ties are represented by thick edges, while weak ties are represented by thin edges

Figure 2 presents network exemplars to illustrate the measures of efficiency, constraint, and closeness centrality. In graph A, ego has high efficiency, because there is no redundancy in the connections. If you removed any edge, the connected alter would be completely isolated from the rest of the network, with no alternative path. In the complete graphs (B and C), there are lots of redundancies in the connections, so efficiency for ego is low. In graph A, constraint on ego is low because ego is connected to many alters which are only connected to each other through ego. In graph B, constraint on ego is high, because the alters are all connected to each other, so ego is only in contact with a single cluster. In graph C, constraint on ego is even higher, because all of ego’s alters have stronger ties to each other than to ego, and thus, ego is only in contact with a single highly connected cluster. In graphs A and B, ego has high closeness centrality, since there is a short path between it and every alter. In graph C, ego has low closeness centrality. Its ties to alters are weak, so the distance between ego and any alter is high.

### Exploratory analysis

We conducted post hoc logistic regression analysis to explore the relationship between support network mean age and RSS, adjusted for respondent age and non-Hispanic white race/ethnicity. When support network mean age was categorized (18–29, 30–39, 40–49, 50 +), the predicted probability of RSS was lower when support network mean age was 50 or above compared to less than 50 (0.27 vs. 0.47; reverse Helmert contrast *χ*^2^ = 7.48, *p* = 0.0062, Bonferroni adjusted *p* = 0.0186). Similarly, we examined the relationship between support network proportion Hispanic and RSS. After examining the distribution of proportion Hispanic, we created a binary indicator to compare majority Hispanic (50% or greater) with majority non-Hispanic. In logistic regression analysis including non-Hispanic white race/ethnicity and support network mean age as covariates, majority Hispanic support network was associated with lower probability of RSS (marginal probability 0.29 vs. 0.47; OR = 0.44, 95% CI 0.23–0.87). Likewise, adjusted for IDU network mean age, having a majority Hispanic support or IDU network had a protective effect on HCV positivity (marginal probability 0.14 vs. 0.37; OR = 0.25, 95% CI 0.11–0.60). Most of the Hispanic participants (70%) in the sample identified as Mexican–American, and this group had a lower HCV positivity prevalence (12.5%) than other Hispanics, and a higher proportion of Hispanic IDU and support network members.

### Sensitivity analysis

When missing alter ties were treated as absent, the RSS model selected 6 additional variables, while the equipment sharing, backloading, and HCV models were the same as with imputation. When cases with missing tie data were excluded, the RSS model selected five additional variables, the equipment sharing model omitted injection network efficiency and included IDU degree, and the HCV model omitted support network gender heterogeneity. The backloading model was the same as with imputation.

## Discussion

Our study examined associations of injection, sex, and support network characteristics with HCV infection and risky injection practices. We identified several network characteristics associated with HCV status, RSS, and equipment sharing, including both injection and support network composition variables. RSS and equipment sharing were also associated with certain measures of injection network structure.

### Injection network composition

Gender and sexual relationships were clearly important factors for all risk behaviors. Previous research has noted the association between having a sex partner who is also an injection partner (i.e., multiplexity) and greater likelihood of sharing syringes [33–37]. In this study, simply having a mixed gender injection network (high heterogeneity or low homophily) was also associated with increased probability of injection risk behavior and was the only predictor associated with backloading. There may be a tendency for women who inject drugs to rely on a more experienced male IDU partner to prepare the shot as well as purchase the drugs, even outside of a sexual relationship.

Previous research has shown that young PWID are more likely to engage in risky injection behavior compared to older PWID [34, 38, 39]. While the respondent’s age was not among the selected variables, the average age of the injection network members was negatively associated with equipment sharing. That is, regardless of the respondent’s own age, injecting with young PWID was associated with a greater probability of sharing equipment. While older age is associated with less risky behavior, it is also associated with a greater likelihood of HCV infection simply by accumulation over time. Hispanic ethnicity of the IDU network was associated with a lower likelihood of HCV infection, but was not associated with reported risk behavior. This is consistent with epidemiological evidence of lower HCV prevalence in US Hispanic populations generally, especially Mexican-Americans [2, 40], and highlights the importance of ethnic mixing for exposure risk.

Our previous research found greater injection risk behavior among young PWID who were transient across urban and suburban areas [15, 41]. Here, we similarly found equipment sharing was more likely among PWID with high injection network residence heterogeneity. That is, PWID with injection networks spanning Cook County and suburban areas outside of Cook County were more likely to report equipment sharing. This is a unique finding that may or may not generalize to other populations, but has implications locally for harm reduction efforts to prevent HCV. Sharing injection equipment other than syringes poses a significant risk for HCV infection [42, 43]. Among PWID with less exposure to harm reduction messages, the risks of equipment sharing may be less salient than those of syringe sharing [44].

### Injection network structure

Previous studies have found that syringe sharing is associated with network size and density [11, 19, 45–48]. In this study, we found that low injection network efficiency was associated with greater probability of both RSS and equipment sharing, and efficiency was negatively correlated with both network size and density. Low efficiency reflects a network that is both large and dense, while a high efficiency network is either small, or large and low density. Taking into account the strength of ties gives greater weight to close relationships. In this sample, the valued measure of efficiency, which takes into account the strength of ties, was more strongly associated with RSS, while the binary measure better predicted equipment sharing. PWID with large, dense networks may be optimal candidates for training as peer educators (PE) to reduce their own risk and to disseminate harm reduction information to their peers [49–51]. Evidence suggests that PE training is at least moderately effective for reducing risk behavior [49, 52–54], but it is intensive and therefore costly [55], yet little attention has been given to the selection of PE candidates to maximize outcomes.

Other structural variables found to be associated with risk behavior in our study include (valued) closeness centrality, which was negatively associated with RSS and (valued) constraint negatively associated with equipment sharing. These two measures are significantly positively correlated (*r* = 0.30, *p* < 0.0001). In an egocentric network, closeness centrality is the reciprocal of the average length of the shortest paths between the ego and each alter, rescaled to range from 0 to 1. Thus, high closeness centrality reflects stronger relationships. It may seem counterintuitive that PWID with stronger ties to their injection network were less likely to report RSS, when at the dyad level PWID are more likely to share syringes with those closer to them [15, 56]. This highlights the importance of distinguishing between dyadic effects and collective network influences and suggests that PWID with a mix of weak and strong ties in their network may be more likely to engage in RSS than those with consistently strong relationships.

We found high constraint was associated with a lower probability of equipment sharing; this was the strongest association with equipment sharing next to non-Hispanic white race/ethnicity. Constraint measures the extent to which the ego is connected to alters, who are in turn connected to one another, and is highly correlated with tie density. The more alters are connected to one another, the more the ego’s behavior may be constrained, as alters can interact with each other without having to go through the ego. The association between constraint and equipment sharing is likely due to selective behavior of the alters having closer ties to one another than to ego.

### Support network composition

Interestingly, having older support network members was protective for RSS. Older support network members are likely to be parents and other relatives. Young PWID who are lacking this type of support network may be more likely to engage in risky behavior. A majority Hispanic support network was also associated with a lower likelihood of both RSS and HCV, regardless of the respondent’s own ethnicity, though Hispanic participants were more likely to have a majority Hispanic support network. Variability in ethnicity of support and IDU networks may also account for differences among Hispanic subgroups, such and Mexican-Americans and Puerto Ricans. A better understanding of network factors that protect Hispanic PWID might yield novel ideas for reducing risk among non-Hispanic PWID.

A mixed gender support network was also protective for HCV. For equipment sharing, having more support network members who lived in Cook County was protective, suggesting that PWID who migrate into the urban center from outer suburbs may be more likely to engage in risky injection behavior. This relates to the finding noted above that IDU network residence heterogeneity was associated with equipment sharing and highlights the need to extend harm reduction efforts geographically.

### Limitations

Over ten percent of respondents had missing network tie data, for which we used imputation. Our sensitivity analysis indicated that imputation of tie data resulted in a more parsimonious model for RSS and made little difference for equipment sharing or backloading. We used lasso regression to identify a set of likely predictors from a large set of variables. This type of analysis does not allow for statistical inference. The predicted strength of the effects is given by the penalized regression coefficients, which are standardized coefficients, but no standard errors or p values are computed. Variable selection is data driven with the goal of prediction rather than explanation. However, this is not necessarily a weakness [57–59]. The results of predictive modeling can stimulate investigation and discovery of causal mechanisms. An integrated approach considering both explanation and prediction can lead to more robust models with greater generalizability [58].

## Conclusions

As in previous research, race/ethnicity, age, gender mixing, and injection network size and density were important influences on injection risk behavior. RSS, but not equipment sharing, was associated with multiplex IDU–sex partner relationships. The associations of IDU and support network geography with equipment sharing highlight the need to extend harm reduction efforts beyond urban areas. We also saw that support network composition may affect injection risk behavior. Greater understanding of these influences may provide important insights to strengthen the benefits of harm reduction. In considering the probability of HCV transmission, it is important to consider both individual risk behaviors and the settings and network structures that promote propagation. In our sample of young urban and suburban PWID, we report on these factors, many of which for the first time.

Harm reduction efforts were successful in significantly reducing HIV among PWID in the USA in the 1990s [60–63]; however, evidence for the impact of harm reduction strategies in reducing HCV transmission among PWID is mixed [64–67]. A recent review showed that SSPs alone have a lower impact in North America compared to Europe, but more positive results were found for combination strategies (SSP and opioid substitution therapy). Because HCV is transmitted much more efficiently than HIV, harm reduction interventions must be scaled up considerably to have an impact. Network-based strategies may help us to scale up more efficiently. Our study elucidated a number of network composition (e.g., age, gender, sexual relationship, ethnicity), network structure (e.g., efficiency, closeness centrality, constraint), and geographic variables (e.g., transience) that were associated with syringe sharing. Given the alarming increases in HCV among US PWID, harm reduction programs that employ network-based strategies may augment impact in the USA to achieve outcomes similar to those in Europe [67]. These may include network-based harm reduction strategies (e.g., provision of the number of syringes by SSPs based on injection network size) or more targeted outreach to high-risk groups (e.g., transient PWID). Given that the USA lags behind other high-income countries in progress toward meeting WHO’s HCV eliminations goals [68], future research could examine the impact of these strategies in regional microelimination of HCV [69].

## Supplementary Information

**Additional file 1. Table S1: **Definitions of network measures

**Additional file 2. Table S2:**Additional statistics for network measures

## Data Availability

The datasets analyzed during the current study are available from the corresponding author on reasonable request subject to a data use agreement.

## Abbreviations

CV: Cross-validation
HCV: Hepatitis C virus
IDU: Injection drug use
MSE: Mean squared error
PWID: People who inject drugs
RSS: Receptive syringe sharing
SSP: Syringe services program
