# Supplementary material for: Egocentric network characteristics of people who inject drugs in the Chicago metro area and associations with hepatitis C virus and injection risk behavior
Source: Harm Reduct J. 2022 Jun 2;19:58. doi: 10.1186/s12954-022-00642-4 (PMC9161656; doi:10.1186/s12954-022-00642-4)
Supplement: Supplementary file 1 — Additional file 1. Table S1: Definitions of network measures [file 12954_2022_642_MOESM1_ESM.docx]

**TABLE S1: Network measures definitions**

| **Measure** | **Definition** |
| --- | --- |
| Degree | the number of connections a node has |
| Tie density | the number of ties in the network divided by the number of possible ties |
| Homophily | the percentage of ego’s alters that have the same value of an attribute (e.g. age, gender, race, residence); a measure of the tendency for like to be connected to like in the graph |
| Heterogeneity | measured by the normalized Blau’s index which quantifies the probability that two members randomly selected from a group belong to different categories; reaches its minimum value (*0*) when all individuals are classified in the same category |
| Effective size | a measure of non-redundant connections; if a node is connected to nodes which are in turn connected with each other, there is a greater redundancy, and thus the effective size will be smaller |
| Efficiency | effective size divided by the actual size (degree) |
| Constraint | measures the extent to which ego is strongly connected to alters which are in turn strongly connected to ego’s other alters |
| Hierarchy | measures the extent to which constraint is concentrated on a single alter |
| Mean multiplexity | the average number of distinct relationship types that tie an ego with its alters |
| Average distance | the average length of the shortest path between an ego and each of its alters |
| Global clustering coefficient | the percentage of triplets of nodes where all three nodes are connected to each other |
| Local clustering coefficient | measures how close a node’s neighborhood is to being a clique (i.e., each neighbor of the node is connected to every other neighbor) |
| Average local clustering | the average value of local clustering for each node in the graph |
| Betweenness centrality | measures how often a node appears on the shortest path between two other nodes; it can be viewed as a measure of control, because if a node has high betweenness centrality, any two nodes that interact with each other are likely to pass through it |
| Closeness centrality | the average length of the shortest path between a node and every other node; a node with high closeness centrality is close to every node in the graph |
| Centralization | quantifies the degree to which the graph as a whole is centralized. This paper uses Freeman centralization, which measures how central the most central node is compared with all other nodes. It ranges from 0 for the least centralized possible graph (a complete graph) to 1 for the most centralized possible graph (a star graph). Betweenness and closeness centralization are based on the corresponding measures of node centrality. |
| Modularity | represents the extent to which the graph can be partitioned into distinct groups, with strong ties between members of the same group and weak or few ties to members of a different group. Modularity was computed by finding the network partition that maximizes Newman’s Q. |
