# Supplementary material for: Egocentric network characteristics of people who inject drugs in the Chicago metro area and associations with hepatitis C virus and injection risk behavior
Source: Harm Reduct J. 2022 Jun 2;19:58. doi: 10.1186/s12954-022-00642-4 (PMC9161656; doi:10.1186/s12954-022-00642-4)
Supplement: Supplementary file 2 — Additional file 2. Table S2:Additional statistics for network measures [file 12954_2022_642_MOESM2_ESM.docx]

| **Table S2: Additional statistics for injection, support and sexual network measures (n=269)** | | | | | | | | | | | | | |
| --- | --- | --- | --- | --- | --- | --- | --- | --- | --- | --- | --- | --- | --- |
|  | **Injection** | | | | **Support** | | | | **Sexual†** | | | |  |
| **Variable** | **Mean** | **SD** | **Min** | **Max** | **Mean** | **SD** | **Min** | **Max** | **Mean** | **SD** | **Min** | **Max** |  |
| alter age variability (SD) | 5.51 | 4.72 | 0 | 30 | 7.53 | 9.18 | 0 | 38 | 1.56 | 3.45 | 0 | 28 |  |
| global clustering | 0.68 | 0.37 | 0 | 1 | 0.51 | 0.48 | 0 | 1 | 0.20 | 0.36 | 0 | 1 |  |
| avg. clustering coef. (bin.) | 0.71 | 0.37 | 0 | 1 | 0.52 | 0.48 | 0 | 1 | 0.20 | 0.37 | 0 | 1 |  |
| avg. clustering coef. (val.) | 0.48 | 0.29 | 0 | 1 | 0.4 | 0.4 | 0 | 1 | 0.14 | 0.27 | 0 | 1 |  |
| local clustering coef. (bin.) | 0.61 | 0.38 | 0 | 1 | 0.49 | 0.47 | 0 | 1 | 0.17 | 0.35 | 0 | 1 |  |
| local clustering coef. (val.) | 0.43 | 0.3 | 0 | 1 | 0.39 | 0.4 | 0 | 1 | 0.12 | 0.26 | 0 | 1 |  |
| between centralization (bin.) | 0.18 | 0.3 | 0 | 1 | 0.09 | 0.26 | 0 | 1 | 0.21 | 0.38 | 0 | 1 |  |
| between centralization (val.) | 0.41 | 0.32 | 0 | 1 | 0.28 | 0.38 | 0 | 1 | 0.29 | 0.42 | 0 | 1 |  |
| betweeness centrality (bin.) | 0.19 | 0.3 | 0 | 1 | 0.1 | 0.26 | 0 | 1 | 0.21 | 0.38 | 0 | 1 |  |
| betweeness centrality (val.) | 0.33 | 0.34 | 0 | 1 | 0.24 | 0.37 | 0 | 1 | 0.26 | 0.41 | 0 | 1 |  |
| effective size (bin.) | 1.93 | 1.33 | 1 | 9 | 1.23 | 0.58 | 1 | 5 | 1.50 | 1.12 | 1 | 9 |  |
| effective size (val.) | 2.53 | 1.6 | 1 | 9 | 1.46 | 0.78 | 1 | 6 | 1.59 | 1.16 | 1 | 9 |  |
| modularity (bin.) | 0.02 | 0.04 | 0 | 0 | 0 | 0.02 | 0 | 0 | 0.00 | 0.02 | 0 | 0 |  |
| modularity (val.) | 0.03 | 0.06 | 0 | 0 | 0.01 | 0.03 | 0 | 0 | 0.00 | 0.03 | 0 | 0 |  |
| race heterogeneity | 0.25 | 0.24 | 0 | 1 | 0.11 | 0.2 | 0 | 1 | 0.11 | 0.20 | 0 | 1 |  |
| residence heterogeneity | 0.12 | 0.19 | 0 | 1 | 0.1 | 0.2 | 0 | 1 | 0.09 | 0.19 | 0 | 1 |  |
| employment heterogeneity | 0.23 | 0.22 | 0 | 1 | 0.16 | 0.22 | 0 | 1 | 0.10 | 0.19 | 0 | 1 |  |
| heterogeneity = Blau's index; bin = binary; val = valued; CC = Cook county | | | | | | | | | | | | | |
| † N = 216 (53 have no sex partners) | | | | | | | | | | | | |  |
